# Practical Hints

**Published:** 1861-07

**Authors:** J. D. White


Practical Hints.—By *J. D. White.*—Tidiness of the dentist is one of the first things which it is his duty to study, and yet it would seem, from many circumstances, that the subject does not receive much attention. Writers have not given it as much consideration as many small mechanical matters which any one's own judgment would properly decide upon. There is a kind of law regulating the tidiness or manner of serving a patient, while in the presence of a dentist, as any other thing we do in life; hence there ought to be some kind of rule laid down, or understood, for the consideration of the dental student, and such detailed hints given as to afford him some kind of guide to be governed by, else he may run from one extreme to another before he knows what he is about, and become disgusting by his own uncultivated inclinations. *Manners* on the part of the dentist, when he is about his patient at the operating chair, is as essential as proper behavior at a dinner-table; and we are sure a great deal has been said by authors of large abilities on that subject. We do not pretend to hold ourselves up as without fault, or to dictate rules and regulations to others, but to refer to some things which would seem to strike the mind as self-evident, yet do not receive proper attention. A dentist in full practice has not the time for drawing-room formalities or all the courtesies of morning calls, nor does any one of common sense expect it; his office and waiting-rooms are places of business, and the majority of persons who call upon him are business people, or in haste from one point of duty to another; and it is not uncommon in these days of railways and steamboats, that well-bred persons commit the most uncivil and annoying interruptions at the dentist's office, for want of time to do better; so in like manner, the dentist, to make time for the patient in hand, is obliged to treat very summarily a patient making a first call, and incurs much censure by it; but good sense on both sides will make in such cases every allowance. It is better for the dentist so to act as to meet the good sense of the intelligent, than to waste time to prevent the ignorant or exacting from finding fault. Biography is said to teach better, perhaps, than anything else, how the good and great have achieved their most noble purposes in life; and while straight and hard lines seem to have marked their career, it is remarkable to see how every act is filled in with attention to the smallest things incident to their every duty.

By knowing others' faults, we should learn to correct our own. But to some details. It is essential that a dentist should have everything in his office to afford comfort and convenience to his patients. His chair should be comfortable and clean ; he should place a large, clean towel over the head-piece of his chair, so as to extend a little way down the back ; this should be removed as soon as it is in the least soiled, and in these days of *youthful-looking hair*, the changing will become frequently necessary ; to place the patient's head against the plush of the head-rest of the chair, is not allowable. It is necessary that every patient should be furnished with a clean napkin and glass of tepid water—a little cologne is desirable, but some few persons can not tolerate it. The head of the patient should be so placed as not to touch the breast of the operator while operating, as it soils his dress, and is uncomfortable to the patient, especially in warm weather. The rule which some dentists practice on, that the patient must endure every *inconvenience* for the time being for the *convenience* of tho dentist, is not good practice, or just in any sense. Whistling and humming tunes in the presence of the patient is vulgar in the extreme. Every dentist should even learn how to *breathe* while operating, because it is true that the breath is not always free from some taint in any one, and where it is habitually bad, it is very unfortunate. A great deal of annoyance may be avoided by the operator breathing through his nose when his face is turned toward his patients while operating or in close proximity to them, or abstaining from speaking while in that attitude. A dentist to hold his mouth open and breathe full in the face of his patients while operating, is enough to suffocate them ; and if there is the slightest taint of anything on his breath, it is intolerable.

A lady patient visited us a short time since from a great distance, leaving a good dentist ; it was with difficulty we could wait on her, and we remarked how useless it was to put herself to such inconvenience when she had so good a dentist near her own home. "Oh, I got tired of the stench of old coffee-grounds breathed in my face all the time," was her reply.

It is useless to enumerate the many articles of diet which affect the breath, which a dentist should avoid during operating hours. Tobacco and liquors in any form, as a matter of course, must be abstained from by every well-bred dentist, before approaching a patient, and every attempt to hide their effects by artificial means makes the evil worse and the suspicion greater. If such habits have been contracted by any one before he adopts dentistry as a profession, it is most unfortunate.

Clean hands may be said to be one of the most important matters connected with the dentist's duties. Besides the necessity of having every convenience to wash his hands frequently, he should study how to prevent them from becoming offensive or soiled. The saliva of some patients becomes very unpleasant when exposed to the air, and when it gets on the hands, it soon renders them offensive; hence we always use a small finger napkin on the lips of the patient, to answer the double purpose of preventing the instruments from chaffing them, and keeping our fingers dry; we also keep a lock of cotton in the left hand, between the fingers, to receive the decay as it is removed from a tooth, to avoid getting it in contact with the fingers, as there is nothing so difficult to remove as the fetor of the decay of a tooth when it gets into the skin. We were present once when a dentist of distinction was operating, and saw him remove the decay from a tooth and wipe it on his finger, which disgusted us very much. The use of scented soap does not effectually remove such stench. We never use highly-flavored soap for the hands, as that in itself is eventually disgusting. Clean, inodorous soap is preferable. The nails on the fingers must be kept reasonably short, and clean.—*Dental Cosmos.*

